# An international literature-based dataset on metallic trace element contamination in kitchen garden plants

**DOI:** 10.1038/s41597-025-05085-7

**Published:** 2025-05-03

**Authors:** Laure Genies, Céline Laurent, Stéphanie Ouvrard, Camille Dumat, Franck Marot, Corinne Hulot, Christophe Schwartz, Géraldine Bidar

**Affiliations:** 1 Université de Lille, IMT Nord Europe, Université d’Artois, JUNIA, ULR 4515 - LGCgE, Laboratoire de Génie Civil et géo-Environnement, F-59000 Lille, France; 2 Université de Lorraine, INRAE, LSE, F-54000 Nancy, France; 3 Dynafor, INRAE Auzeville & Toulouse INP, Auzeville-Tolosane, France; 4 ADEME, Agence de la Transition Ecologique, Service Sitésol, 49004 Angers, France; 5 Ineris, Parc technologique Alata, BP 2, F-60550 Verneuil-en-Halatte, France

**Keywords:** Environmental impact, Plant sciences

## Abstract

Urban agriculture is an attractive solution to counter artificialization and densification of (sub)urban areas, offering environmental, socio-economic and health benefits. Nevertheless, the transfer of metallic trace elements (MTE) from soil, water or air to crops raises concerns about potential health risks for consumers in urban environments. Assessing environmental suitability is therefore essential for safe food production. To support this, the BAPPET dataset compiles data on MTE concentrations in 90 edible plants commonly grown in kitchen gardens. Built from 528 international studies, the dataset also describes MTE content in the environment and key parameters influencing MTE transfer to plants: (i) Plant information: species, variety, cultivation conditions, harvesting details; (ii) Growing media: MTE concentrations in soil, air and water, agronomic soil parameters affecting MTE availability; (iii) Experimental context: MTE contamination sources, methodological details. With over 29,000 analyses, the BAPPET dataset provides a comprehensive data collection for the comparison of site-specific results in environmental diagnoses and for the construction and validation of transfer models.

## Background & Summary

Since the first industrial revolution, cities have been constantly expanding in industrialized and developing countries. This urban sprawl has generated close proximity between inhabitants and industries as well as an increase of urban brownfield. The enthusiasm for reintroducing nature in cities, creating social links, and the desire to eat healthily and inexpensively has led to brownfields reclaiming and rehabilitating into community gardens or urban farms, and encouraging householders to cultivate their own kitchen garden. This increases the risk of people’s exposure to pollutants^1,2^, notably through the consumption of contaminated vegetables grown in these urban soils.

Metallic trace elements (MTE) contamination of vegetable gardens in (sub)urban soils has a wide range of origins^3^ whether related to urbanization (heating, traffic) or farming practices (phytosanitary products and fertilizers, irrigation water). Created in 2007, the BAPPET dataset (*BAse de données sur la contamination des Plantes Potagères par les Eléments Traces métalliques* - dataset on the contamination of kitchen garden crops by metallic trace elements) was designed to support such health risks assessments related to the consumption of vegetables grown in potentially contaminated environments. The BAPPET dataset gathers bibliographic data on the MTE contamination of several vegetable species and on growth media in different soil pollution contexts, based on studies conducted in 65 countries. This concerns arsenic (As), cadmium (Cd), cobalt (Co), chromium (Cr), copper (Cu), mercury (Hg), molybdenum (Mo), nickel (Ni), lead (Pb), antimony (Sb), selenium (Se), thallium (Tl), vanadium (V) and zinc (Zn).

First published in 2008 on the website of the French Agency of Ecological Transition ADEME (https://librairie.ademe.fr/), BAPPET was updated for the first time in 2012 with new bibliographic references and a user manual. A decade later (2023), a second update both bibliographic and technical was carried out on the BAPPET dataset. In addition to always being usable on the ADEME open data portal (https://data.ademe.fr/datasets/base-bappet), the dataset is also freely available in table form^4^ and located in Recherche Data Gouv, an open data repository, compliant with “FAIR” principles. On this shared data platform, users can find a dataset similar to BAPPET but dedicated to data collection on a set of organic molecule concentrations in several kitchen garden crop types and associated soils (named BAPPOP^5^).

## Methods

### Overview

**Fig. 1:**
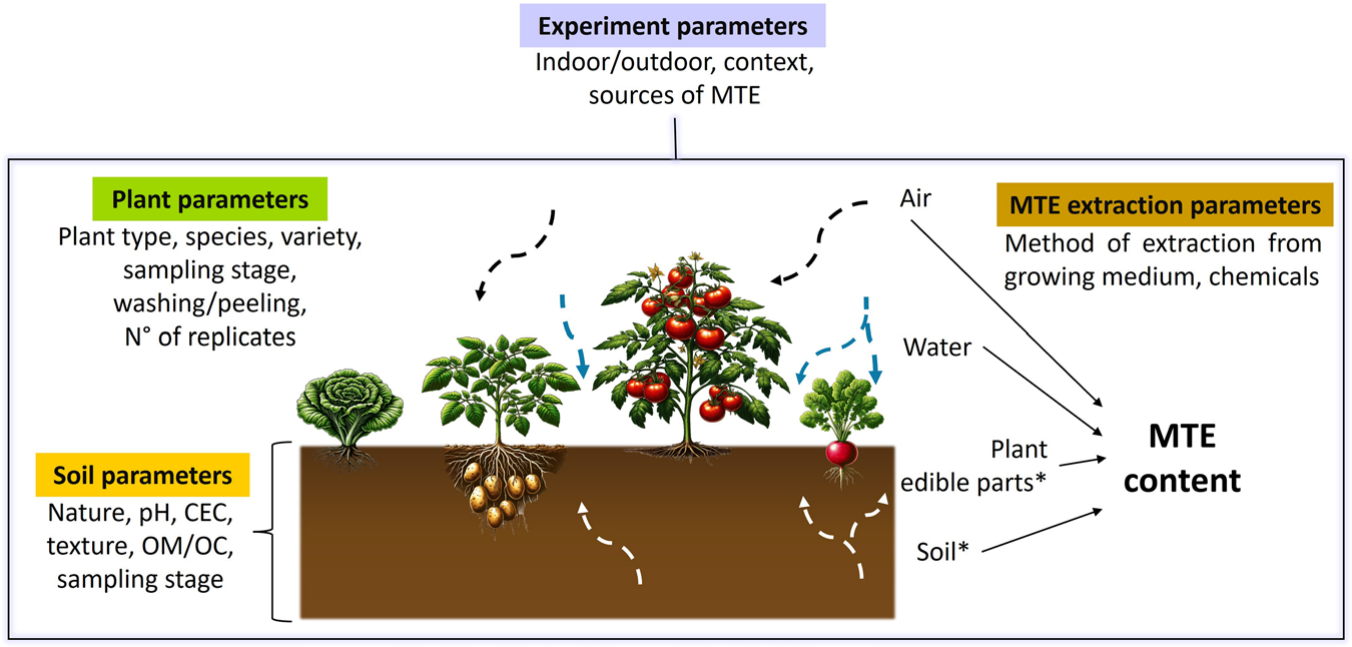
Transfer of MTE in kitchen garden crops: overview of parameters recorded in the BAPPET dataset. Dashed arrows represent transfer of MTE from soil, water and air. Asterisks indicate that MTE content is reported at least for the plant and the soil compartments for each experiment of the BAPPET dataset.

The BAPPET dataset was built on data extracted from published experimental results of MTE transfer into kitchen garden plants, distributed into 10 classes (Tables 1, 2). MTE concentrations in edible parts of 90 plant species and in associated soils are stored in this dataset as well as data related to the main soil physico-chemical parameters known to influence MTE (phyto-)availability (Fig. 1). Each row in the BAPPET dataset represents a single analysis, defined as the measurement of one MTE in a specific plant species (or variety or edible organ), grown under defined experimental conditions, in a soil with specific physico-chemical characteristics, and using a particular MTE extraction method. A total of 69 parameters arranged in columns (detailed below) describe over 29,000 analyses in the BAPPET dataset.

**Table 1:** Plant list and data distribution in leaf-, fruit-, root- and tuber vegetables.

Plant class	Plant common name and data distribution
Leaf vegetables 2,147 experiments (32.7%)	Lettuce (*Lactuca sativa*), 38.9%
	Chinese cabbage, pakchoi/petsai (*Brassica rapa* (*subsp. chinensis* or *pekinensis*)), 15.4%
	Spinach (*Spinacia oleracea*), 14.9%
	Cabbage (*Brassica oleracea*), 12.3%
	Swiss chard (*Beta vulgaris*), 5.6%
	Water spinach (*Ipomoea aquatica*), 3.5%
	Leek (*Allium porrum*) 2.8%
	Celery (*Apium graveolens*), 2.0%
	Edible amaranth (leaves) (*Amaranthus sp*.), 1.4%
	Belgian/White endive or Witloof chicory (*Cichorium intybus*), 1.1%
	Endive, Curly endive, Escarole or broad-leaved endive (*Cichorium endivia*), 0.9%
	Red cabbage (*Brassica oleracea* var. *capitata f. rubra*), 0.4%
	Curly kale (*Brassica oleracea* var. sabellica), 0.3%
	Arugula (*Eruca vesicaria subsp. sativa*), 0.2%
	Edible chrysanthemum (*Glebionis coronaria* ou *Chrysanthemum coronarium*), 0.1%
	Fennel (*Foeniculum vulgare* var. *dulce*), 0.1%
	Watercress (*Nasturtium officinale*), 0.1%
	Brussels sprouts (*Brassica oleracea* var. *gemmifera*), 0.05%
	Nettle (*Urtica dioica*), 0.05%
	Chinese kale (*Brassica oleracea* var. *alboglabra*), 0.05%
Fruit vegetables 1,239 experiments (18.9%)	Tomato (*Solanum lycopersicum*), 38.4%
	French beans or green beans (*Phaseolus vulgaris*), 15.6%
	Hot pepper (*Capsicum frutescens*), 10.4%
	Zucchini or marrow (*Cucurbita pepo*), 10.1%
	Eggplant (*Solanum melongena*), 9.5%
	Pepper (*Capsicum annuum*), 9.2%
	Cucumber (and gherkin) (*Cucumis sativus*), 3.8%
	Pumpkin and winter squash (*Cucurbita sp*.), 2.3%
	Okra (*Abelmoschus esculentus*), 0.5%
	Sugar snap pea (*Pisum sativum*), 0.2%
	Pumpkin (*Cucurbita maxima*), 0.1%
Root vegetables 1,139 experiments (17.3%)	Radish (*Raphanus sativus*), 44.6%
	Carrot (*Daucus carota*), 40.6%
	Beetroot (*Beta vulgaris*), 9.1%
	Celeriac (*Apium graveolens*), 2.8%
	Turnip (*Brassica rapa*), 2.8%
Tuber vegetables 856 experiments (13.0%)	Potato (*Solanum tuberosum*), 96.3%
	Sweet potato (*Ipomoea batatas*), 2.3%
	Jerusalem artichoke (*Helianthus tuberosus*), 1.4%

**Table 2:** Plant list and data distribution in fruits, stem-, bulb- and inflorescence vegetables, and herbs.

Plant class	Plant common name and data distribution
Fruits 302 experiments (4.6%)	Grape, grapevine (*Vitis vinifera*), 31.8%
	Apple (*Malus domestica*), 13.2%
	Plum (*Prunus domestica*), 10.3%
	Strawberry (*Fragaria*), 9.9%
	Pear (*Pyrus communis*), 6.3%
	Fig (*Ficus carica*), 6.0%
	Peach (*Prunus persica*), 5.6%
	Raspberry (*Rubus idaeus*), 3.0%
	Cherry (sour) (*Prunus cerasus*), 2.3%
	Lemon (*Citrus sinensis*), 2.3%
	Melon (*Cucumis melo*), 2.3%
	Blackberry (*Rubus fruticosus*), 2.0%
	Chestnut (*Castanea sp*.), 1.0%
	Mulberry (*Morus sp*.), 1.0%
	Persimmon (*Diospyros kaki*), 1.0%
	Blueberry (*Vaccinium myrtillus*), 0.7%
	Walnut (*Juglans regia*), 0.7%
	Gooseberry (*Ribes rubrum*), 0.3%
	Watermelon (*Citrullus lanatus*), 0.3%
Stem vegetables 255 experiments (3.9%)	Leek (*Allium porrum*), 78.4%
	Swiss chard (*Beta vulgaris*) 14.6%
	Kohlrabi (*Brassica oleracea* var. *gongylodes*), 2.7%
	Celery (*Apium graveolens*) 2.3%
	Rhubarb (*Rheum rhaponticum*), 2%
	Asparagus lettuce (*Lactuca sativa*), 1.5%
Bulb vegetables 198 experiments (3.0%)	Onion (bulb) (*Allium cepa*), 80.3%
	Garlic (*Allium sativum*), 14.1%
	Shallot (*Allium ascalonicum*), 5.6%
Herbs 173 experiments (2.6%)	Onion (leaves), spring onion (*Allium cepa*), 22.5%
	Parsley (*Petroselinum crispum*), 20.8%
	Coriander (*Coriandrum sativum*), 14.5%
	Rosemary (*Salvia rosmarinus*), 11.0%
	Mint (*Mentha spicata*), 9.2%
	Basil (*Ocimum basilicum*), 6.4%
	Welsh onion or japanese bunching onion (*Allium fistulosum*), 5.2%
	Thyme (*Thymus vulgaris*), 4.0%
	Chives (*Allium schoenoprasum*), 1.7%
	Garlic chives or Chinese chives (*Allium tuberosum*), 1.7%
	Oregano (*Origanum vulgare*), 1.7%
	Dill (*Anethum graveolens*), 1.2%
Legumes 167 experiments (2.5%)	Dry bean, kidney bean, flageolet (*Phaesolus vulgaris*), 32.1%
	Green or garden pea (*Pisum sativum*), 23.8%
	Sweet Corn (*Zea mays*), 21.4%
	Cowpea (*Vigna unguiculata*), 10.7%
	Broad bean or Faba bean (*Vicia faba*), 4.2%
	Chickpea (*Cicer arietinum*), 3.6%
	Mung bean (*Vigna radiata*), 3.0%
	Wrinkled pea, Field pea (*Pisum sativum*), 1.2%
Inflorescence vegetables 89 experiments (1.4%)	Cauliflower (*Brassica oleracea* var. *botrytis*), 57.3%
	Globe artichoke (*Cynara scolymus*), 27.0%
	Broccoli (*Brassica oleracea* var. *italica*), 15.7%

An experiment is defined as a group of such analyses conducted on the same plant species/variety and organ, grown on a soil with specific physico-chemicals characteristics under the same experimental condition, but possibly involving multiple MTE and/or MTE extraction method. Over 6,500 experiments (or soil/plant pairings) are recorded in the BAPPET dataset.

### Data sources

Two types of sources dealing with MTE contamination of kitchen garden plants were used for collection of the BAPPET data: (i) scientific papers from a peer-reviewed process (487 references) and (ii) the’grey literature’ (*e.g*. experimental or diagnostic reports - 42 references). Since BAPPET’s creation in 2007 and during its two updates (2012 and 2023), the search for bibliographic resources has been carried out on the Web of Sciences (WOS) research engine. During the last update, more than 16,000 articles published between January 2012 and December 2022 dealing with transfer of MTE to plants were added. Given the large number of references, more specific keywords were used: {(arsenic OR cadmium OR cobalt OR chromium OR copper OR mercury OR molybdenum OR nickel OR lead OR selenium OR thallium OR zinc OR metal) AND (Vegetable OR Fruit Or Edible) AND (Transfer OR Uptake OR Accumulation) AND Soil} reducing the number of references to 3,295 on the WOS in October 2022.

## Data selection

**Fig. 2:**
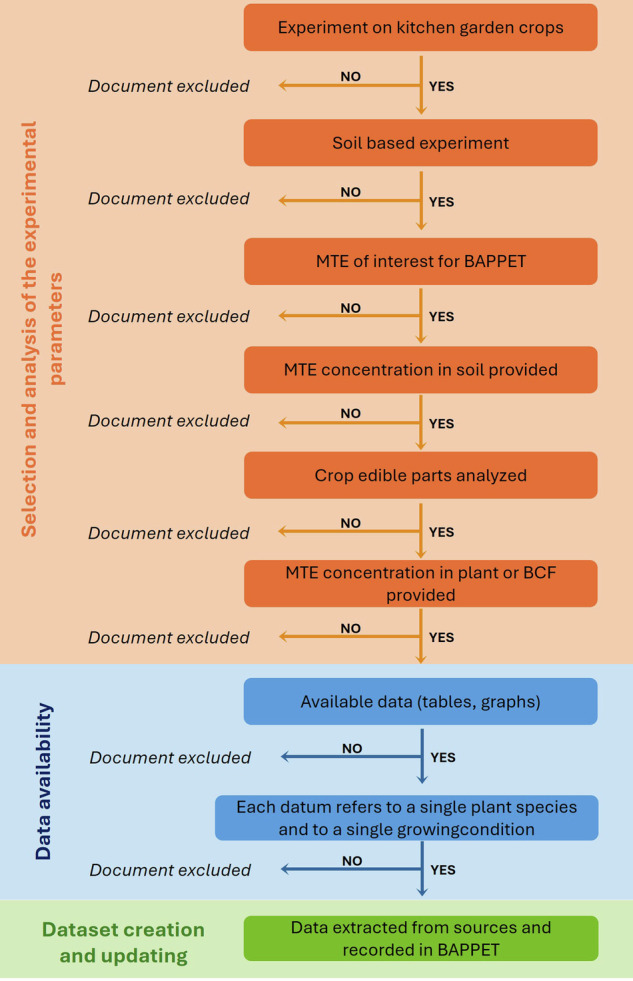
Decision-making flowchart for data implementation in the BAPPET dataset. BCF stands for bioconcentration factor.

**Fig. 3:**
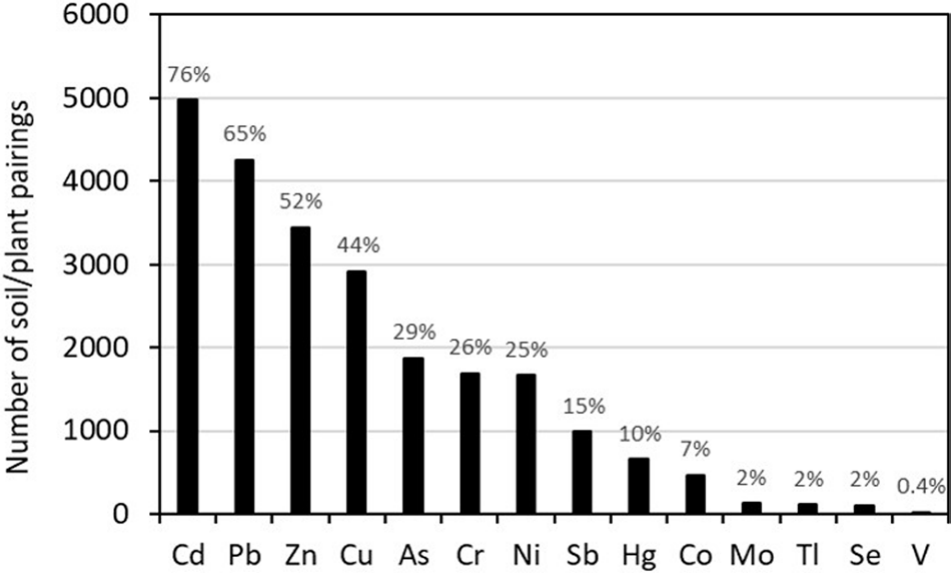
Distribution of soil/plant pairings across the different MTE listed in the BAPPET dataset. The labels indicate the percentage of soil/plant pairings for which the MTE has been entered.

After recovering the scientific sources from WOS, relevant scientific publications from which data were extracted were selected according to a screening process (Fig. 2). First, publications must be dedicated to experiments on kitchen garden crops. Although cereals are excluded from selection, an exception has been made for sweetcorn which is relatively common in kitchen gardens. Then metallic elements investigated in publications must be one of the 14 elements listed in BAPPET, *i.e*. As, Cd, Co, Cr, Cu, Hg, Mo, Ni, Pb, Sb, Se, Tl, V, Zn. Cadmium (Cd), lead (Pb) and zinc (Zn) are the most represented metal in the dataset with more than 50% of the soil/plant pairings dedicated to the study of their transfer (Fig. 3). Only experiments carried out on soils are selected. Those in hydroponic conditions or with direct use of substrates such as compost or biosolids without soil are excluded from the selection. Another condition for publications to be included in the dataset is that they must be able to provide data on concentrations of metal pollutants in plants and soils or bioconcentration factors (BCF). These results must be presented species by species and concern only edible parts of the plant. Thus, data from a mix of different plant species or from the entire plant as well as those derived from models are rejected from the selection. To enable relevant comparisons with user data, publications contributing to BAPPET must explicitly state whether results are reported in fresh or dry weight.

At the end of this selection process, 528 publications were recorded in BAPPET including 37 experimental reports from French public institutes, 2 site diagnostic reports coming from private companies and 3 thesis manuscripts.

### Description of data extracted and standardization

Various parameters influencing the transfer of MTE from the growth medium to the plant as well as information on the context of experimentations, sampling and analysis procedures were extracted from the selected publications and recorded in BAPPET (Fig. 1). To enable comparisons between heterogeneous published results, data standardization was performed. All these parameters and standardization process are described in this section.

The two first columns of the dataset inform on the **experiment number** and bibliographic **reference code**. A unique experiment number is assigned to each soil/plant pairing defining as a single plant species or variety or edible part grown in one single condition. A unique reference code, made with the three first letters of author’s name and year of publication, is assigned to each source document.

The following parameters concern the nature of the analysed **MTE** and the **MTE speciation**. Normalized data entered from a current list of 14 MTE referred to by their chemical symbol: As, Cd, Co, Cr, Cu, Hg, Mo, Ni, Pb, Sb, Se, Tl, V, Zn. The MTE ‘speciation’ column is a free text entry indicating the oxidation number (*e.g.* Cr(VI)) or the complex formula (*e.g.* Na_2_SeO_3_) of the compound.

The next 26 parameters describe the studied plant and associated metadata. First, the **category** of the edible part analysed is indicated (‘Plant type’), using a standardized list of 10 plant types based on the consumed part: leaf-, fruit-, root-, tuber-, stem-, bulb-, inflorescent-vegetable, legume, fruit and herb (Tables 1, 2). Leaf vegetable category is the most represented in the dataset accounting for a third of the data (Table 1).

Then, plant identification is provided through **common names** in **French** and in **English** (‘Species (en)’), as well as scientific **Latin names** (‘Species (lat)’). Since the same kitchen garden crop can be referred to by multiple vernacular and botanical names among different source documents, plant names were standardized employing the nomenclature from the European and Mediterranean Plant Protection Organization (EPPO) Global Database^6^. Taxonomic rank below species (**subspecies, variety, cultivar** entered as free text under ‘Plant variety’) are also included when specified in the source document.

Other information concerns plant sampling with i) the **sampling stage**, indicating the duration of cultivation (in weeks or months) before sampling of the plant, ii) the **edible organ** analysed, iii) whether the plant had reached **maturity** (Yes or No) according to the authors’ indications, and iv) and **sampling effort**, i.e. number of samples analysed. Information on the **plant preparation** (washing/peeling) before MTE analysis is also recorded.

Descriptive statistics of **plant MTE concentration** in edible part analysed, expressed in mg of element per kg of plant (mg/kg) after unit conversion (when necessary) are available in the dataset including **mean,** **standard deviation,** **minimum,** **maximum,** **median,** **geometric standard deviation**. A column (‘DW or FW’) indicates whether MTE concentrations are expressed by dry or fresh plant weigh. Detection and quantification limits of the apparatus (LOD/LOQ, given in mg/kg) are included when they were provided in the source document. If calculated in the source document as concentration of MTE in the edible part of the plant divided by the concentration of MTE in the soil, **bioconcentration factors** are also recorded in the dataset with corresponding statistics: **mean,** **standard deviation,** **minimum** and **maximum**. These parameters (plant MTE concentration and BCF) correspond to free decimal number entry according to information extracted from the source document. In the case of graphical data, the online tool WebPlotDigitizer^7^ was used to transform it into digital data.

The **dry matter content** of the plant is also included in the dataset when provided, after systematically unit conversion in % (‘DW (%)’).

The dataset also compiles **model equation** and **coefficient of determination** R² when MTE concentrations in plant are modelled with soil parameters in the original study.

The next 13 parameters concern characteristics of soil that means plant growing media. The BAPPET dataset first provides the general **description of soil and subsoil** based on details given in the source document including nature, texture and land uses. More precisely, proportion of **clay sized particles** is detailed with **mean, minimum** and **maximum** expressed in %. Average amounts of **sand and silt size particles** are also provided in %. Values originally expressed in g/kg in the source documents were converted in % for each class of particles.

Another characteristic on the soil of each experimentation available in the dataset is the **pH** described by **average**, **minimum** and **maximum** values. **Soil organic matter** and **organic carbon** amounts are also mentioned in the dataset, both expressed in % after unit conversion when necessary. Additionally, the BAPPET dataset includes soil **Cationic Exchange Capacity** (CEC) expressed in cmol^+^/kg. CEC values originally expressed in meq/100 g in the source documents were converted accordingly.

Finally, information on **soil sampling time** is provided through normalized data indicating whether sampling occurred i) before crop planting, ii) during crop growing, iii) at crop harvesting, and iv) after crop harvesting.

The fifth part of the dataset gathers data describing the medium identified as the potentially source of plant contamination. The first parameter specifies **the nature of the investigated medium** (i.e. soil, irrigation water, groundwater, runoff water, or air) and the **unit of MTE concentrations** measured in this medium specified in brackets. MTE concentrations are expressed (after unit conversion of the original data when necessary) in mg/kg or µg/kg for soil, mg/L for water, and mg/m^3^ for air.

The two next columns (‘Extraction’ and ‘Extractants’) inform respectively on the **MTE pool** investigated (*i.e.* total, pseudo total, available, bound to a specific chemical compound of soil or leached) and the **used methods and chemical extractants** to access these MTE pools.

As for plants, the **concentration of MTE** in the investigated medium are statistically described in the dataset with **mean,** **standard deviation,** **minimum,** **maximum** and **median**. The online tool WebPlotDigitizer^7^ was also used to extract numerical data from graphs.

When provided in the original study, the **detection and quantification limit** of the apparatus (LOD/LOQ, expressed in mg/kg) used to analyze MTE concentration in the medium is also included in the dataset.

The next columns cluster informs on the background of the experimentation. First details provide information on the **experiment type** according to these following standardized modalities: indoor or outdoor (in field or kitchen garden or pots), with or without shelter.

The **environmental context** described in the source document is also reported in the dataset. Identified contexts include agricultural, industrial, not anthropized, urban, and artificial environments. In some case, studies may be associated with multiple environmental contexts simultaneously.

Further information is then provided on the **origin of MTE**, describing the type of activities and sources accountable for the presence of MTE in the growing environment. This parameter, tightly linked to the environmental context, includes agricultural, industrial, not anthropogenic, urban and artificial sources. Additionally, a specific modality is included for past or ongoing mining activities which are reported in several studies as sources of MTE contamination.

This section on experimentation background concludes with a free-text **comment** column, providing any additional information on experimental design which may help users to understand the experiment.

The bibliographic references of the source documents are given directly in the last part of the dataset for each experiment recorded, except for confidential data from private companies. The first two columns of this section list the first author, and either the second author or‘*et al*.’ for publications with more than two authors. The **title** of the source document is then given as well as the **year** of the publication, the name of the **journal** where the document is published, the **volume**, issue **number** and **pages**. The **DOI** affiliated to the source document is also indicated when available.

Additional information include the **country** where the experiment was conducted and, finally, the **nature** of the source document categorized using normalized code with 'ART' for peer-reviewed scientific article, 'EDR' for environmental diagnose report, 'EXP' for public experimental report and 'THESE' for thesis manuscript.

## Data Records

The latest release of the dataset is archived on Recherche Data Gouv^4^ as a single table consisting of raw data. A complete dictionary of all recorded parameters and a full list of the current 90 plant species documented in BAPPET are also provided. Any future updating of the dataset will be released in the same format, allowing compatibility with other software and systems, under the same reference.

An Excel file, containing raw data and a multicriteria research form, is also freely available on the ADEME open data portal (https://data.ademe.fr/datasets/base-bappet). This research form has been coded in VBA (Visual Basic for Application) and aims to facilitate the filtering of data. Regular update of the dataset will integrate new scientific advances in this field and hopefully increase numbers of soil/plant pairings for some metal(loid)s such as Sb, Hg, Co, Mo, Tl, Se and V (Fig. 3).

## Technical Validation

No modifications were made to the original data, except for (i) the Latin and vernacular names of the kitchen garden species tested, which were adjusted to match the nomenclature of the EPPO Global Database^6^, and (ii) the conversion of values into the same unit for all studies. Regarding the data collected in scientific published papers, it is assumed that the quality of the methodology and analysis (MTE content in soil and plant, soil parameters) was checked in the peer-review process. The ’grey literature’ includes studies involving French public institutions (such as ADEME, INERIS, Geoderis, French Chambers of Agriculture, French universities) or consulting and engineering companies specialised in the management of Polluted Sites and Soils). These studies are supported by various guidelines drawn up with the aim of optimizing good experimentation practice^8–12^.

An automatic calculation of the Z-Score is available only in the version of the BAPPET dataset hosted on the ADEME open data portal (https://data.ademe.fr/datasets/base-bappet), which includes a search macro. This metric is computed dynamically based on the subset of data selected by the user through the research form. It indicates the distance (in standard deviations) between each sample’s value and the mean of all other samples filtered according to the user’s criteria. The Z-Score is not provided in the raw dataset stored in Recherche Data Gouv repository^4^.

To prevent heterogeneity and/or misspelling during data entry into BAPPET, operators used a VBA entry form with drop-down menus. For numerical inputs, data were extracted from PDF files using Excel’s data import function, when it was possible, to avoid typing errors. The error rate for digitising graphs from sources using WebPlotDigitizer^7^ is estimated between 0 and 16% (mean error 1.7% ± 2.1; median error 0.85%; n = 219 values from 14 different papers).

## Data Availability

No custom code was used to generate or process the data described in the paper.
